# Lord–Wingersky Algorithm Version 2.5 with Applications

**DOI:** 10.1007/s11336-021-09785-y

**Published:** 2021-07-27

**Authors:** Sijia Huang, Li Cai

**Affiliations:** 1 Indiana University Bloomington, Bloomington, IN USA; 2 University of California, Los Angeles (UCLA), CRESST, 300 Charles E. Young Dr. North, GSEIS Building, Los Angeles, CA 90095-1522 USA

**Keywords:** hierarchical item factor model, summed score, subscore, bifactor model

## Abstract

Item response theory scoring based on summed scores is employed frequently in the practice of educational and psychological measurement. Lord and Wingersky (Appl Psychol Meas 8(4):453–461, 1984) proposed a recursive algorithm to compute the summed score likelihood. Cai (Psychometrika 80(2):535–559, 2015) extended the original Lord–Wingersky algorithm to the case of two-tier multidimensional item factor models and called it Lord–Wingersky algorithm Version 2.0. The 2.0 algorithm utilizes dimension reduction to efficiently compute summed score likelihoods associated with the general dimensions in the model. The output of the algorithm is useful for various purposes, for example, scoring, scale alignment, and model fit checking. In the research reported here, a further extension to the Lord–Wingersky algorithm 2.0 is proposed. The new algorithm, which we call Lord–Wingersky algorithm Version 2.5, yields the summed score likelihoods for all latent variables in the model conditional on observed score combinations. The proposed algorithm is illustrated with empirical data for three potential application areas: (a) describing achievement growth using score combinations across adjacent grades, (b) identification of noteworthy subscores for reporting, and (c) detection of aberrant responses.

## Introduction

Generalizing the seminal Lord–Wingersky (1984) algorithm to other settings has been a regular topic in item response theory (IRT) research since its initial publication more than 35 years ago. Also well known in the Rasch modeling community (Andersen, 1972; Gustafsson, 1980), this simple recursive algorithm’s wide-reaching impact in psychometrics is impressive to behold. For example, Hanson (1994), Thissen et al. (1995), as well as von Davier and Rost (1995), were among the first to expand the algorithm to polytomous IRT models. Chen and Thissen (1999) derived an item calibration algorithm based on summed scores. Thissen and Wainer’s (2001) influential text on test scoring presented extensive methods for handling mixed-format tests, including an approach to handle score combinations (Rosa et al., 2001) that heavily influenced our thinking in the study reported here. Orlando et al. (2000) applied the Lord–Wingersky algorithm to illustrate summed score-based test linking, another area consistently of interest to psychometricians (e.g., Zeng & Kolen, 1995; Thissen et al., 2011). Orlando and Thissen (2000) proposed a solution to the item fit testing problem with a slight alteration of the original Lord–Wingersky algorithm. Li and Cai (2018) further extended the algorithm to create more accurate distributional approximations for test statistics sensitive to latent variable distributional assumptions. Stucky (2009), and independently Kim (2013), developed the weighted version of the algorithm wherein the item scores can take non-integer values.

Cai (2015) extended the algorithm to the case of hierarchical item factor models, specifically the two-tier model (Cai, 2010b). He named it Lord–Wingersky algorithm 2.0. In brief, a two-tier model consists of *M* primary latent dimensions ($$\varvec{\eta })$$ and *N* specific latent dimensions ($$\xi _{n}$$, $$n=1, \ldots N)$$. The specific dimensions are independent conditional on the primary latent dimensions. Each item can load on at most one specific latent dimension, creating *N* non-overlapping *item clusters*. The item bifactor model (Gibbons & Hedeker, 1992), a member of the two-tier family (where $$M=1)$$, has experienced particular theoretical and empirical success recently (see Cai et al., 2011; Reise et al., 2007, 2018; Reise, 2012). In addition, the standard correlated-traits MIRT model (Reckase, 2009) and the testlet response theory model (Wainer et al., 2007) are constrained versions of the two-tier model. The two-tier structure permits the implementation of a dimension reduction technique (Rijmen, 2009) for computationally efficient maximum marginal likelihood parameter estimation with quadrature.

The dominating insight of Cai (2015) is that the non-overlapping item clusters are exchangeable conditional on the primary latent dimensions. In the original Lord–Wingersky algorithm, the items and their item scores are the basic building blocks. In the Lord–Wingersky algorithm 2.0, item clusters take the place of items and become the fungible units of model building and computation. Once again, dimension reduction can efficiently handle the numerical integration with quadrature. The algorithm yields summed score to scaled score conversions for the primary dimension(s), along with other associated statistical indices, with $(M+1)$-fold integration regardless of the total number of factors in the model.

The present study extends the Lord–Wingersky algorithm 2.0. The new algorithm (Lord–Wingersky algorithm 2.5) uses patterns of item cluster summed scores instead of the overall summed score in Lord–Wingersky algorithm 2.0. Specifically, the item cluster summed score patterns are combinations of the observed score from one cluster and the summed score of the rest of the item clusters. It reduces to cluster score combinations when there are only two item clusters. It is worth noting here that the idea of using observed scores patterns to score individuals in unidimensional IRT is not new (e.g., Rosa et al., 2001). The algorithm proposed in this study generalizes this idea to scenarios where the underlying IRT models are hierarchical item factor models. Lord–Wingersky algorithm 2.5 leads to multidimensional posteriors of the primary latent dimension(s) with each specific latent dimension. The posterior probability of each observed score combination is a natural by-product. We illustrate applications of the proposed algorithm with three examples.

In the first example, we fit a longitudinal IRT model and use the Lord–Wingersky algorithm 2.5 to enhance the growth interpretation of score scales across adjacent grades in an operational large-scale English language proficiency assessment program, all without having to set a “vertical” scale. Second, the bivariate posteriors and score combination probabilities are used to facilitate the decision-making on subscore reporting. Finally, we construct posterior high-density region (HDR) for observed score combinations to help detect aberrant responses.

## Lord–Wingersky Algorithm 2.0

We briefly review Cai’s (2015) Lord–Wingersky algorithm 2.0 to establish notation.

With no loss of generality, consider a bifactor model with *N* specific latent dimensions, wherein each $\xi _{n}$ is measured by $I_{n}$ dichotomously scored items, and $n=1,\ldots ,N$. Let the prior (population) distribution of the general dimension $\eta$ be denoted $h(\eta )$. To avoid notational clutter, instead of assuming conditional independence of the prior distributions of the specific dimensions $g(\xi _{n}\mathrm {\vert }\eta )$ on $\eta$, we will assume, again with no loss of generality, fully independent specific dimensions. In other words, we shall write $g\mathrm {(}\xi _{n}\mathrm {)}$ as the prior of $\xi _{n}$. Define $T_{i}\left( 1\vert {\eta , \xi _{n}}\right)$ as the item response function of the *i*th item $(i=1,\ldots I_{n})$ in cluster *n*, such that

1 $$\begin{aligned} T_{i}\left( 1\vert {\eta , \xi _{n}}\right) =\frac{1}{1+\exp \left[ -\left( c_{i}+a_{i}^{0}\eta +a_{i}^{n}\xi _{n} \right) \right] } \end{aligned}$$

where $a_{i}^{0}$ and $a_{i}^{n}$ are the primary latent dimension and specific latent dimension item slopes, respectively, and $c_{i}$ is the item intercept. The item parameters are assumed to be known and fixed, usually from a calibration study.

### Stage I

In the first stage of Lord–Wingersky algorithm 2.0, for each item cluster, the within-cluster summed score likelihoods are accumulated over the latent space spanned by the primary latent dimension and the specific latent dimension. Let $P_{i}^{n}(x\vert \eta , \xi _{n})$ denote the likelihood of summed score *x* after including the *i*th item in item cluster *n* in the recursive computation to be described below. Consider the *n*th item cluster, the algorithm initializes with the first item by starting the likelihood of summed score 0 $P_{1}^{n}\left( 0\vert {\eta ,\xi _{n}}\right)$ at the item response probabilities $T_{1}\left( 0\vert {\eta ,\xi _{n}}\right)$, and $P_{1}^{n}\left( 1\vert {\eta ,\xi _{n}}\right) =T_{1}\left( 1\vert {\eta ,\xi _{n}}\right)$. Then, the second item is added, resulting in three available summed scores: 0, 1, and 2. The corresponding summed score likelihoods after adding item 2 are:

2 $$\begin{aligned}&P_{2}^{n}\left( 0\vert {\eta ,\xi _{n}}\right) =P_{1}^{n}\left( 0\vert {\eta ,\xi _{n}}\right) T_{2}\left( 0\vert {\eta ,\xi _{n}}\right) ,\nonumber \\&P_{2}^{n}\left( 1\vert {\eta ,\xi _{n}}\right) =P_{1}^{n}\left( 1\vert {\eta ,\xi _{n}}\right) T_{2}\left( 0\vert {\eta ,\xi _{n}}\right) +P_{1}^{n}\left( 0\vert {\eta ,\xi _{n}}\right) T_{2}\left( 1\vert {\eta ,\xi _{n}}\right) \nonumber \\&P_{2}^{n}\left( 2\vert {\eta ,\xi _{n}}\right) =P_{1}^{n}\left( 1\vert {\eta ,\xi _{n}}\right) T_{2}\left( 1\vert {\eta ,\xi _{n}}\right) . \end{aligned}$$

After this, each of the remaining items in item cluster *n* is included in the computation to form the desired within-cluster summed score likelihoods. Specifically, in step $i (2<i\le I_{n})$ of the recursive algorithm, the *i*th item is added as follows:

3 $$\begin{aligned}&P_{i}^{n}\left( 0\vert {\eta ,\xi _{n}}\right) =P_{i-1}^{n}\left( 0\vert {\eta ,\xi _{n}}\right) T_{i}\left( 0\vert {\eta , \xi _{n}}\right) \nonumber \\&P_{i}^{n}\left( x\vert {\eta ,\xi _{n}}\right) =P_{i-1}^{n}\left( x\vert {\eta ,\xi _{n}}\right) T_{i}\left( 0\vert {\eta , \xi _{n}}\right) +P_{i-1}^{n}\left( {x-1}\vert {\eta ,\xi _{n}}\right) T_{i}\left( 1\vert {\eta , \xi _{n}}\right) \nonumber \\&P_{i}^{n}\left( i\vert {\eta ,\xi _{n}}\right) =P_{i}^{n}\left( {i-1}\vert {\eta ,\xi _{n}}\right) T_{i}\left( 1\vert {\eta , \xi _{n}}\right) . \end{aligned}$$

The middle equation in (3) is repeated over values of *x* between 1 and $i-1$.

To avoid notational clutter, let $P_{n}\left( s_{n}\vert {\eta \mathrm {,}\xi _{n}}\right) =P_{I_{n}}^{n}\left( s_{n}\vert {\eta ,\xi _{n}}\right)$ denote the likelihood associated with the within-cluster summed score $s_{n}=0,\ldots ,I_{n}$, after all $I_{n}$ items in cluster *n* have been added according to the recursions defined in Eq. (3). At this point, an extra step is performed. The specific latent dimension, $\xi _{n}$, is integrated out, leaving the summed score likelihoods solely a function of the primary latent dimension, $\eta$. For simplicity, we can approximate this integral with rectangular quadrature:

4 $$\begin{aligned} P_{n}\left( s_{n}\vert \eta \right) =\int {P_{n}\left( s_{n}\vert {\eta \mathrm {,}\xi _{n}}\right) } g\left( \xi _{n} \right) d\xi _{n}\approx \sum \limits _{q=1}^Q {P_{n}\left( s_{n}\vert {\eta \mathrm {,}Y_{q}}\right) W_{n}(Y_{q})}, \end{aligned}$$

where *Q* is the number of quadrature points, $Y_{q}$ the *q*th quadrature point, and $W_{n}(Y_{q})$ is the corresponding quadrature weight, computed as normalized ordinates of $g\left( \xi _{n} \right)$.

### Stage II

At the end of the first stage, available to us are *N* sets of within-cluster summed score likelihoods $\left\{ P_{n}\left( s_{n}\vert \eta \right) ;s_{n}=0,\ldots ,I_{n} \right\}$, for $n=1,\ldots ,N$. These quantities depend only on the primary latent dimension $\eta$. Each item cluster can now be treated as if it were a polytomous item with $I_{n}+1$ categories, and the “item scores” range from 0 to $I_{n}$.

Denote $L_{n}(s\vert \eta )$ as the likelihood of summed score *s* after adding item cluster *n* to the existing summed score likelihoods in the recursive computation described below. Let $S_{n}$ be the maximum obtainable summed score after adding item cluster *n*. In our context when the items are all dichotomous, $S_{n}=\sum \nolimits _{j=1}^n I_{j}$ . Obviously $S_{N}$ would be the maximum summed score. At this point, the standard Lord–Wingersky algorithm for polytomous items can be applied.

Let $L_{1}\left( s_{1}\vert \eta \right) =P_{1}\left( s_{1}\vert \eta \right) , \mathrm {\forall } s_{1}=0,\ldots ,I_{1}$, for the purpose of initialization. Then in step $n (2<n\le N)$, the likelihoods $P_{n}\left( s_{n}\vert \eta \right)$ from item cluster *n* are added to the likelihoods from previous step to form the desired summed score likelihoods. For each possible summed score $0\le s\le S_{n}$, we let

5 $$\begin{aligned} L_{n}\left( s\vert \eta \right) =\mathop {\sum }\limits _{s_{n-1}=0}^{S_{n-1}} \mathop {\sum }\limits _{s_{n}=0}^{I_{n}} {L_{n-1}\left( s_{n-1}\vert \eta \right) P_{n}\left( s_{n}\vert \eta \right) } \mathbf {1}_{s}\left( s_{n-1}+s_{n} \right) , \end{aligned}$$

where $\mathbf {1}_{s}(s_{n-1}+s_{n})$ is an indicator function and takes the value of 1 if $s_{n-1}+s_{n}=s$ and 0 otherwise. Equation (5) essentially involves the booking keeping for a pair of scores $s_{n-1}$ (from all item clusters added previously) and $s_{n}$ (from the current item cluster) that adds up to the summed score *s*. When all *N* item clusters are included $L_{N}(s\vert \eta )$—or simply $L(s\vert \eta )$ to reduce clutter—contains the summed score likelihoods for the primary dimensions for $0\le s\le S_{N}$.

### Posterior Summaries

Recall that $h\mathrm {(}\eta \mathrm {)}$ is the prior distribution of the primary latent dimension. The normalized posterior of $\eta$ associated with summed score *s* is

6 $$\begin{aligned} p\left( \eta \vert s\right) =\frac{L\left( s\vert \eta \right) h\left( \eta \right) }{p\left( s \right) } \end{aligned}$$

where $p\left( s \right)$ is the (marginal) probability of summed score *s*:

7 $$\begin{aligned} p\left( s \right) =\int {L\left( s\vert \eta \right) h(\eta )d\eta } \approx \sum \limits _{q=1}^Q {L\left( s\vert Y_{q}\right) W\left( Y_{q} \right) }, \end{aligned}$$

and *Q* rectangular quadrature points $$X_{q} $$ are used to approximate the posterior, with $$W\left( X_{q} \right) $$ the normalized ordinates of $$h(\eta )$$. The posterior mean $$E\left( \eta \vert s\right) $$ and posterior variance $$Var\left( \eta \vert s\right) =E\left( \eta ^{2}\vert s\right) - E^{2}\left( \eta \vert s\right) $$ are useful summaries, where

8 $$\begin{aligned}&E\left( \eta \vert s\right) =\frac{1}{p\left( s \right) }\int {\eta L\left( s\vert \eta \right) h\left( \eta \right) } d\eta \approx \frac{1}{p\left( s \right) }\sum \limits _{q=1}^Q {X_{q}L\left( s\vert X_{q}\right) W\left( X_{q} \right) }, \nonumber \\&E\left( \eta ^{2}\vert s\right) =\frac{1}{p\left( s \right) }\int {\eta ^{2}L\left( s\vert \eta \right) h\left( \eta \right) } d\eta \approx \frac{1}{p\left( s \right) }\sum \limits _{q=1}^Q {X_{q}^{2}L\left( s\vert X_{q}\right) W\left( X_{q} \right) }. \end{aligned}$$

A normal approximation of the posterior based on the posterior mean and variance often works quite well even when the number of items is moderate. The posterior mean can be used as the summed score-based IRT scaled score estimate and the posterior variance as the error variance estimate for the scaled score. The marginal probability $p\left( s \right)$ itself can be useful either as a model-based (pre-operational) estimated of the expected summed score group probability or as an aid in IRT model fit checking.

## Lord–Wingersky Algorithm 2.5

### General Approach

Recall the bifactor model with *N* specific latent dimensions defined in Sect. 2. Each of the *N* item clusters includes $I_{n}$ items. The Lord–Wingersky algorithm 2.0 is focused on obtaining the posterior distribution of the primary dimension $\eta$, conditioned on the overall summed score. The specific latent dimensions are integrated out at the end of Stage I (see Sect. 2.1). In the proposed algorithm, we obtain bivariate posteriors of the primary latent dimension $\eta$ and the specific latent dimension $\xi _{n}$.

Instead of the overall summed score, each bivariate posterior is conditioned on a pair of scores. We continue to use $s_{n}$ to denote the summed scores from item cluster *n*, where $s_{n}=0,\ldots ,I_{n}$, and introduce here new notation for the *rest score* $s_{(n)}$, i.e., the summed score from all clusters except item cluster *n*. Let $S_{(n)}=\sum \nolimits _{j=1, j\ne n}^N I_{j}$ be the maximum summed score from the rest of the item clusters so $s_{(n)}=0,\ldots ,S_{(n)}$. Cai (2015) in fact alluded to the possibility of using the summed vs. rest score combination $(s_{n},s_{(n)})$, but stopped shy of actually computing the bivariate posterior, as we now outline below.

### Stage I

In the first stage, for each item cluster, the within-cluster summed score likelihoods are accumulated over the space spanned by $\eta$ and $\xi _{n}, n=1,\ldots N$, just as in Lord–Wingersky algorithm 2.0. At the end of this stage, we retain and store the likelihoods for the primary dimension $\left\{ P_{n}\left( s_{n}\vert \eta \right) \mathrm {; }s_{n}=0,\ldots ,I_{n} \right\} , \forall n=1,\ldots ,N$. The critical added requirement is that we also retain and store all the within-cluster summed score likelihoods $\left\{ P_{n}\left( s_{n}\vert {\eta ,\xi _{n}}\right) \mathrm {; }\,s_{n}=0,\ldots ,I_{n} \right\} , \forall n=1,\ldots ,N$. In a quadrature representation of the likelihoods, at most $Q\times Q$ floating point values are stored per cluster, per score, if *Q* quadrature points per dimension are used.

### Stage II

We will now cycle through the item clusters to compute the desired bivariate posteriors. In general, for item cluster *n*, we wish to construct bivariate posteriors for $\eta$ and $\xi _{n}$. Recall that the other item clusters do not depend on $\xi _{n}$, so we proceed by treating the cluster summed score likelihood values $P_{1}\left( s_{1}\vert \eta \right) ,\ldots ,P_{n-1}\left( s_{n-1}\vert \eta \right) , P_{n+1}\left( s_{n+1}\vert \eta \right) ,\ldots ,P_{N}(s_{N}\vert \eta )$ as though they were polytomous items that depend on $\eta$. The standard Lord–Wingersky algorithm can now be applied readily to produce item cluster *n*’s rest score likelihoods $R_{n}\left( s_{(n)}\vert \eta \right)$, for $s_{(n)}=0,\ldots , S_{(n)}$. In other words, the recursions work in exactly the same manner as Sect. 2.2, except that we omit the likelihood contributions from $P_{n}\left( s_{n}\vert \eta \right)$.

The rest score likelihoods $R_{n}\left( s_{(n)}\vert \eta \right)$ are then combined with the summed score likelihoods from item cluster *n*, $P_{n}\left( s_{n}\vert {\eta ,\xi _{n}}\right)$, $s=0,\ldots ,I_{n}$, as well as the prior distributions for $\eta$ and $\xi _{n}$, to yield the bivariate posterior distributions of $\eta$ and $\xi _{n}$ associated with the summed vs. rest score combination $(s_{n}, s_{(n)})$:

9 $$\begin{aligned} p\left( {\eta ,\xi _{n}}\vert {s_{n}, s_{(n)}}\right) =\frac{P_{n}\left( s_{n}\vert {\eta ,\xi _{n}}\right) R_{n}\left( s_{(n)}\vert \eta \right) g\left( \xi _{n} \right) h\left( \eta \right) }{p\left( s_{n},s_{(n)} \right) }, \end{aligned}$$

where the marginal probability $p\left( s_{n},s_{(n)} \right)$ is

10 $$\begin{aligned} p\left( s_{n},s_{\left( n \right) } \right) =\iint {P_{n}\left( s_{n}\vert {\eta ,\xi _{n}}\right) R_{n}\left( s_{(n)}\vert \eta \right) g\left( \xi _{n} \right) h\left( \eta \right) d\xi _{n}d\eta }. \end{aligned}$$

Again, the posterior above can be easily approximated with rectangular quadrature:

11 $$\begin{aligned} p\left( s_{n},s_{\left( n \right) } \right) \approx \sum \limits _{r=1}^Q \sum \limits _{q=1}^Q {P_{n}\left( s_{n}\vert {X_{r},Y_{q}}\right) R_{n}\left( s_{\left( n \right) }\vert X_{r}\right) W_{n}\left( Y_{q} \right) W(X_{r})}. \end{aligned}$$

Aside from the marginal probability in Eq. (10), other useful summaries of the posterior distribution include the mean vector $\varvec{\mu }$ the covariance matrix ${\varvec{\Sigma }}$, which facilitate a bivariate normal approximation to the posterior that can be quite effective in practice, as we shall demonstrate later. The marginal posterior means $\mu _{0}=E\left( \eta \vert s_{n}, s_{(n)} \right)$ and $\mu _{n}=E\left( \xi _{n}\vert s_{n}, s_{(n)} \right)$, and the error variances and covariance $\sigma _{00}= Var\left( \eta \vert s_{n}, s_{(n)} \right)$, $\sigma _{0n}=Cov\left( \eta ,\xi _{n}\vert s_{n}, s_{(n)} \right)$, and $\sigma _{nn}=Var\left( \xi _{n}\vert s_{n}, s_{(n)} \right)$ provide reasonable point estimates and error (co)variance estimates for all the latent variables in the model. These means and covariance matrix elements can be approximated with quadrature along similar lines as Eq. (11).

### An Illustrative Example

Consider the same hypothetical six-item scale with bifactor structure as discussed in Cai (2015). These six dichotomous items form three item clusters, each consisting of two items. Priors of all four latent dimensions (one primary latent dimension and three specific latent dimensions) are assumed independent and standard normal. Table 1 shows the item parameters and the factor pattern.

**Table 1:** Item parameters of the six-item scale

Item	$a^{0}$	$a^{1}$	$a^{2}$	$a^{3}$	*c*
1	1.2	1.0			$-$ 1.0
2	1.2	1.0			$-$ .6
3	1.0		.8		$-$ .2
4	1.0		.8		.2
5	.8			1.2	.6
6	.8			1.2	1.0

**Table 2:** Accumulating within-in summed score likelihoods for item cluster 1, 2, and 3

	Quadrature grid for $\left( \eta , \xi _{1} \right)$								
*Initializing with item 1 in cluster 1, having two available summed scores*									
$\eta$	$-$ 2	$-$ 2	$-$ 2	$\cdots$	0	$\cdots$	2	2	2
$\xi _{1}$	$-$ 2	$-$ 1	0	$\cdots$	0	$\cdots$	0	1	2
$P_{1}^{1}\left( 0\vert {\eta ,\xi _{1}}\right) =T_{1}\left( 0\vert {\eta ,\xi _{1}}\right)$	.996	.988	.968	$\cdots$	.731	$\cdots$	.198	.083	.032
$P_{1}^{1}\left( 1\vert {\eta ,\xi _{1}}\right) =T_{1}\left( 1\vert {\eta ,\xi _{1}}\right)$	.004	.012	.032	$\cdots$	.269	$\cdots$	.802	.917	.968
*Adding item 2 to the computation*									
$P_{2}^{1}\left( 0\vert {\eta ,\xi _{1}}\right) =P_{1}^{1}\left( 0\vert {\eta ,\xi _{1}}\right) T_{2}\left( 0\vert {\eta ,\xi _{1}}\right)$	.989	.970	.922	$\cdots$	.472	$\cdots$	.028	.005	.001
$P_{2}^{1}\left( 1\vert {\eta ,\xi _{1}}\right) =P_{1}^{1}\left( 0\vert {\eta ,\xi _{1}}\right) T_{2}\left( 1\vert {\eta ,\xi _{1}}\right) +P_{1}^{1}\left( 1\vert {\eta ,\xi _{1}}\right) T_{2}\left( 0\vert {\eta ,\xi _{1}}\right)$	.011	.030	.077	$\cdots$	.433	$\cdots$	.284	.131	.053
$P_{2}^{1}\left( 2\vert {\eta ,\xi _{1}}\right) =P_{1}^{1}\left( 1\vert {\eta ,\xi _{1}}\right) T_{2}\left( 1\vert {\eta ,\xi _{1}}\right)$	.000	.000	.002	$\cdots$	.095	$\cdots$	.688	.864	.947

	Quadrature grid for $\left( \eta , \xi _{2} \right)$								
*Initializing with item 1 in cluster 2, having two available summed scores*									
$\eta$	$-$ 2	$-$ 2	$-$ 2	$\cdots$	0	$\cdots$	2	2	2
$\xi _{2}$	$-$ 2	$-$ 1	0	$\cdots$	0	$\cdots$	0	1	2
$P_{1}^{2}\left( 0\vert {\eta ,\xi _{2}}\right) =T_{1}\left( 0\vert {\eta ,\xi _{2}}\right)$	.978	.953	.900	$\cdots$	.550	$\cdots$	.142	.069	.032
$P_{1}^{2}\left( 1\vert {\eta ,\xi _{2}}\right) =T_{1}\left( 1\vert {\eta ,\xi _{2}}\right)$	.022	.047	.100	$\cdots$	.450	$\cdots$	.858	.931	.968
*Adding item 2 to the computation*									
$P_{2}^{2}\left( 0\vert {\eta ,\xi _{2}}\right) =P_{1}^{2}\left( 0\vert {\eta ,\xi _{2}}\right) T_{2}\left( 0\vert {\eta ,\xi _{2}}\right)$	.947	.887	.773	$\cdots$	.248	$\cdots$	.014	.003	.001
$P_{2}^{2}\left( 1\vert {\eta ,\xi _{2}}\right) =P_{1}^{2}\left( 0\vert {\eta ,\xi _{2}}\right) T_{2}\left( 1\vert {\eta ,\xi _{2}}\right) +P_{1}^{2}\left( 1\vert {\eta ,\xi _{2}}\right) T_{2}\left( 0\vert {\eta ,\xi _{2}}\right)$	.053	.110	.213	$\cdots$	.505	$\cdots$	.213	.110	.053
$P_{2}^{2}\left( 2\vert {\eta ,\xi _{2}}\right) =P_{1}^{2}\left( 1\vert {\eta ,\xi _{2}}\right) T_{2}\left( 1\vert {\eta ,\xi _{2}}\right)$	.001	.003	.014	$\cdots$	.248	$\cdots$	.773	.887	.947

	Quadrature grid for $\left( \eta , \xi _{3} \right)$								
*Initializing with item 1 in cluster 3, having two available summed scores*									
$\eta$	$-$ 2	$-$ 2	$-$ 2	$\cdots$	0	$\cdots$	2	2	2
$\xi _{3}$	$-$ 2	$-$ 1	0	$\cdots$	0	$\cdots$	0	1	2
$P_{1}^{3}\left( 0\vert {\eta ,\xi _{3}}\right) =T_{1}\left( 0\vert {\eta ,\xi _{3}}\right)$	.968	.900	.731	$\cdots$	.354	$\cdots$	.100	.032	.010
$P_{1}^{3}\left( 1\vert {\eta ,\xi _{3}}\right) =T_{1}\left( 1\vert {\eta ,\xi _{3}}\right)$	.032	.100	.269	$\cdots$	.646	$\cdots$	.900	.968	.990
*Adding item 2 to the computation*									
$P_{2}^{3}\left( 0\vert {\eta ,\xi _{3}}\right) =P_{1}^{3}\left( 0\vert {\eta ,\xi _{3}}\right) T_{2}\left( 0\vert {\eta ,\xi _{3}}\right)$	.922	.773	.472	$\cdots$	.095	$\cdots$	.007	.001	.000
$P_{2}^{3}\left( 1\vert {\eta ,\xi _{3}}\right) =P_{1}^{3}\left( 0\vert {\eta ,\xi _{3}}\right) T_{2}\left( 1\vert {\eta ,\xi _{3}}\right) +P_{1}^{3}\left( 1\vert {\eta ,\xi _{3}}\right) T_{2}\left( 0\vert {\eta ,\xi _{3}}\right)$	.077	.213	.433	$\cdots$	.433	$\cdots$	.155	.053	.017
$P_{2}^{3}\left( 2\vert {\eta ,\xi _{3}}\right) =P_{1}^{3}\left( 1\vert {\eta ,\xi _{3}}\right) T_{2}\left( 1\vert {\eta ,\xi _{3}}\right)$	.002	.014	.095	$\cdots$	.472	$\cdots$	.838	.947	.983

**Table 3:** Integrating the specific dimensions $\xi _{2}$ and $\xi _{3}$ out of the summed score likelihoods

	Quadrature grid for $\left( \eta , \xi _{2} \right)$								
$\eta$	$-$ 2	$-$ 2	$-$ 2	$\ldots$	0	$\ldots$	2	2	2
$\xi _{2}$	$-$ 2	$-$ 1	0	$\ldots$	0	$\ldots$	0	1	2
$W_{2}(\xi _{2})$	.054	.244	.403	$\ldots$	.403	$\ldots$	.403	.244	.054
Multiplying cluster 2’s summed score likelihoods by $W_{2}\left( \xi _{2} \right)$									
$P_{2}\left( 0\vert {\eta ,\xi _{2}}\right) W_{2}\left( \xi _{2} \right) =P_{2}^{2}\left( 0\vert {\eta ,\xi _{2}}\right) W_{2}\left( \xi _{2} \right)$	052	.217	.311	$\ldots$	.100	$\ldots$	.006	.001	.000
$P_{2}\left( 1\vert {\eta ,\xi _{2}}\right) W_{2}\left( \xi _{2} \right) =P_{2}^{2}\left( 1\vert {\eta ,\xi _{2}}\right) W_{2}\left( \xi _{2} \right)$	.003	.027	.086	$\ldots$	.203	$\ldots$	.086	.027	.003
$P_{2}\left( 2\vert {\eta ,\xi _{2}}\right) W_{2}\left( \xi _{2} \right) =P_{2}^{2}\left( 2\vert {\eta ,\xi _{2}}\right) W_{2}\left( \xi _{2} \right)$	.000	.001	.006	$\ldots$	.100	$\ldots$	.311	.217	.052

	Quadrature grid for $\eta$								
	$-$ 2		$-$ 1		0		1		2
Leaving cluster’2 summed score as a function of $$\eta $$, by integrating out $$\xi _{2}$$ (summing over $$X_{q})$$									
$P_{2}\left( 0\vert \eta \right) =\sum \limits _{\xi _{2}} {P_{2}\left( 0\vert {\eta ,\xi _{2}}\right) W_{2}\left( \xi _{2} \right) }$	.742		.519		.277		.106		.028
$P_{2}\left( 1\vert \eta \right) =\sum \limits _{\xi _{2}} {P_{2}\left( 0\vert {\eta ,\xi _{2}}\right) W_{2}\left( \xi _{2} \right) }$	.230		.375		.446		.375		.230
$P_{2}\left( 2\vert \eta \right) =\sum \limits _{\xi _{2}} {P_{2}\left( 0\vert {\eta ,\xi _{2}}\right) W_{2}\left( \xi _{2} \right) }$	.028		.106		.277		.519		.742

	Quadrature grid for $\left( \eta , \xi _{3} \right)$								
$\eta$	$-$ 2	$-$ 2	$-$ 2	$\cdots$	0	$\cdots$	2	2	2
$\xi _{3}$	$-$ 2	$-$ 1	0	$\cdots$	0	$\cdots$	0	1	2
$W_{3}(\xi _{3})$	.054	.244	.403	$\cdots$	.403	$\cdots$	.403	.244	.054
Multiplying cluster 3’s summed score likelihoods by $W_{3}(\xi _{3})$									
$P_{3}\left( 0\vert {\eta ,\xi _{3}}\right) W_{3}(\xi _{3})=P_{2}^{3}\left( 0\vert {\eta ,\xi _{3}}\right) W_{3}(\xi _{3})$	.050	.189	.190	$\cdots$	.038	$\cdots$	.003	.000	.000
$P_{3}\left( 1\vert {\eta ,\xi _{3}}\right) W_{3}(\xi _{3})=P_{2}^{3}\left( 1\vert {\eta ,\xi _{3}}\right) W_{3}(\xi _{3})$	.004	.052	.174	$\cdots$	.174	$\cdots$	.062	.013	.001
$P_{3}\left( 2\vert {\eta ,\xi _{3}}\right) W_{3}(\xi _{3})=P_{2}^{3}\left( 2\vert {\eta ,\xi _{3}}\right) W_{3}(\xi _{3})$	.000	.003	.038	$\cdots$	.190	$\cdots$	.337	.231	.054

	Quadrature grid for $\eta$								
	$-$ 2		$-$ 1		0		1		2
Leaving cluster’3 summed score as a function of $\eta$, by integrating out $\xi _{3}$ (summing over $\xi _{3})$									
$P_{3}\left( 0\vert \eta \right) =\sum \limits _{\xi _{3}} {P_{3}\left( 0\vert {\eta ,\xi _{3}}\right) W_{3}(\xi _{3})}$	.469		.302		.166		.077		.029
$P_{3}\left( 1\vert \eta \right) =\sum \limits _{\xi _{3}} {P_{3}\left( 1\vert {\eta ,\xi _{3}}\right) W_{3}(\xi _{3})}$	.364		.396		.364		.285		.192
$P_{3}\left( 2\vert \eta \right) =\sum \limits _{\xi _{3}} {P_{3}\left( 2\vert {\eta ,\xi _{3}}\right) W_{3}(\xi _{3})}$	.166		.302		.469		.638		.779

**Table 4:** Forming the rest score likelihoods (summed score likelihoods for item clusters 2 and 3)

	Quadrature Grid for $\eta$				
	$-$ 2	$-$ 1	0	1	2
*Initializing with cluster 2, having 3 available summed scores*					
$L_{2}\left( 0\vert \eta \right) =P_{2}(0\vert \eta )$	.742	.519	.277	.106	.028
$L_{2}\left( 1\vert \eta \right) =P_{2}(1\vert \eta )$	.230	.375	.446	.375	.230
$L_{2}\left( 2\vert \eta \right) =P_{2}(2\vert \eta )$	.028	.106	.277	.519	.742
*Adding cluster 3’ summed scores to item cluster 1’s rest score likelihoods*					
$R_{1}\left( 0\vert \eta \right) =L_{3}\left( 0\vert \eta \right) =L_{2}(0\vert \eta )P_{3}(0\vert \eta )$	.348	.157	.046	.008	.001
${R_{1}\left( 1\vert \eta \right) =L}_{3}\left( 1\vert \eta \right) =L_{2}\left( 0\vert \eta \right) P_{3}\left( 1\vert \eta \right) +L_{2}\left( 1\vert \eta \right) P_{3}\left( 0\vert \eta \right)$	.378	.319	.175	.059	.012
${R_{1}\left( 2\vert \eta \right) =L}_{3}\left( 2\vert \eta \right) =L_{2}\left( 0\vert \eta \right) P_{3}\left( 2\vert \eta \right) +$ $L_{2}\left( 1\vert \eta \right) P_{3}\left( 1\vert \eta \right) +L_{2}(2\vert \eta )P_{3}(0\vert \eta )$	.220	.337	.339	.214	.088
$R_{1}\left( 3\vert \eta \right) =L_{3}\left( 3\vert \eta \right) =L_{2}\left( 1\vert \eta \right) P_{3}\left( 2\vert \eta \right) +L_{2}(2\vert \eta )P_{3}(1\vert \eta )$	.049	.155	.310	.387	.321
$R_{1}\left( 4\vert \eta \right) =L_{3}\left( 4\vert \eta \right) =L_{2}(2\vert \eta )P_{3}(2\vert \eta )$	.005	.032	.130	.331	.578

**Table 5:** Forming posteriors for score combinations related to item cluster 1

	Quadrature grid for $\left( \eta , \xi _{1} \right)$								
$\eta$	$-$ 2	$-$ 2	$-$ 2	$\cdots$	0	$\cdots$	2	2	2
$\xi _{1}$	$-$ 2	$-$ 1	0	$\cdots$	0	$\cdots$	0	1	2
$W(\eta )$	.054	.054	.054	$\cdots$	.403	$\cdots$	.054	.054	.054
$W_{1}(\xi _{1})$	.054	.244	.403	$\cdots$	.403	$\cdots$	.403	.244	.054
$p\left( {0,0}\vert {\eta ,\xi _{1}}\right) \propto P_{1}(0\vert \eta , \xi _{1})R_{1}\left( 0\vert \eta \right) W(\eta )W_{1}(\xi _{1})$	.0010	.0045	.0070	$\mathrm {\cdots }$	.0035	$\mathrm {\cdots }$	.0000	.0000	.0000
$p\left( {1,0}\vert {\eta ,\xi _{1}}\right) \propto P_{1}(1\vert \eta , \xi _{1})R_{1}\left( 0\vert \eta \right) W(\eta )W_{1}(\xi _{1})$	.0000	.0001	.0006	$\mathrm {\cdots }$	.0032	$\mathrm {\cdots }$	.0000	.0000	.0000
$p\left( {2,0}\vert {\eta ,\xi _{1}}\right) \propto P_{1}(2\vert \eta , \xi _{1})R_{1}\left( 0\vert \eta \right) W(\eta )W_{1}(\xi _{1})$	.0000	.0000	.0000	$\mathrm {\cdots }$	.0007	$\mathrm {\cdots }$	.0000	.0000	.0000
$p\left( {0,1}\vert {\eta ,\xi _{1}}\right) \propto P_{1}(0\vert \eta , \xi _{1})R_{1}\left( 1\vert \eta \right) W(\eta )W_{1}(\xi _{1})$	.0011	.0049	.0077	$\mathrm {\cdots }$	.0134	$\mathrm {\cdots }$	.0000	.0000	.0000
$p\left( {1,1}\vert {\eta ,\xi _{1}}\right) \propto P_{1}(1\vert \eta , \xi _{1})R_{1}\left( 1\vert \eta \right) W(\eta )W_{1}(\xi _{1})$	.0000	.0001	.0006	$\mathrm {\cdots }$	.0123	$\mathrm {\cdots }$	.0001	.0000	.0000
$p\left( {2,1}\vert {\eta ,\xi _{1}}\right) \propto P_{1}(2\vert \eta , \xi _{1})R_{1}\left( 1\vert \eta \right) W(\eta )W_{1}(\xi _{1})$	.0000	.0000	.0000	$\mathrm {\cdots }$	.0027	$\mathrm {\cdots }$	.0002	.0001	.0000
$p\left( {0,2}\vert {\eta ,\xi _{1}}\right) \propto P_{1}(0\vert \eta , \xi _{1})R_{1}\left( 2\vert \eta \right) W(\eta )W_{1}(\xi _{1})$	.0006	.0028	.0045	$\mathrm {\cdots }$	.0259	$\mathrm {\cdots }$	.0001	.0000	.0000
$p\left( {1,2}\vert {\eta ,\xi _{1}}\right) \propto P_{1}(1\vert \eta , \xi _{1})R_{1}\left( 2\vert \eta \right) W(\eta )W_{1}(\xi _{1})$	.0000	.0001	.0004	$\mathrm {\cdots }$	.0237	$\mathrm {\cdots }$	.0005	.0002	.0000
$p\left( {2,2}\vert {\eta ,\xi _{1}}\right) \propto P_{1}(2\vert \eta , \xi _{1})R_{1}\left( 2\vert \eta \right) W(\eta )W_{1}(\xi _{1})$	.0000	.0000	.0000	$\mathrm {\cdots }$	.0052	$\mathrm {\cdots }$	.0013	.0010	.0002
$p\left( {0,3}\vert {\eta ,\xi _{1}}\right) \propto P_{1}(0\vert \eta , \xi _{1})R_{1}\left( 3\vert \eta \right) W(\eta )W_{1}(\xi _{1})$	.0001	.0006	.0010	$\mathrm {\cdots }$	.0237	$\mathrm {\cdots }$	.0002	.0000	.0000
$p\left( {1,3}\vert {\eta ,\xi _{1}}\right) \propto P_{1}(1\vert \eta , \xi _{1})R_{1}\left( 3\vert \eta \right) W(\eta )W_{1}(\xi _{1})$	.0000	.0000	.0001	$\mathrm {\cdots }$	.0218	$\mathrm {\cdots }$	.0020	.0006	.0001
$p\left( {2,3}\vert {\eta ,\xi _{1}}\right) \propto P_{1}(2\vert \eta , \xi _{1})R_{1}\left( 3\vert \eta \right) W(\eta )W_{1}(\xi _{1})$	.0000	.0000	.0000	$\mathrm {\cdots }$	.0048	$\mathrm {\cdots }$	.0049	.0037	.0009
$p\left( {0,4}\vert {\eta ,\xi _{1}}\right) \propto P_{1}(0\vert \eta , \xi _{1})R_{1}\left( 4\vert \eta \right) W(\eta )W_{1}(\xi _{1})$	.0000	.0001	.0001	$\mathrm {\cdots }$	.0100	$\mathrm {\cdots }$	.0004	.0000	.0000
$p\left( {1,4}\vert {\eta ,\xi _{1}}\right) \propto P_{1}(1\vert \eta , \xi _{1})R_{1}\left( 4\vert \eta \right) W(\eta )W_{1}(\xi _{1})$	.0000	.0000	.0000	$\mathrm {\cdots }$	.0091	$\mathrm {\cdots }$	.0036	.0010	.0001
$p\left( {2,4}\vert {\eta ,\xi _{1}}\right) \propto P_{1}(2\vert \eta , \xi _{1})R_{1}\left( 4\vert \eta \right) W(\eta )W_{1}(\xi _{1})$	.0000	.0000	.0000	$\mathrm {\cdots }$	.0020	$\mathrm {\cdots }$	.0087	.0066	.0016

For each dimension, $Q= 5$ equally spaced quadrature points at $-$ 2, $-$ 1, 0, 1, and 2 are used for demonstrate purposes only (practical usage of the algorithm requires much larger values of *Q*). Thus, a $5\times 5$ grid is formed as the direct product of $\eta$ and each of the three specific latent dimensions when appropriate. Summed score likelihoods are evaluated over these grid points.

Tables 2 and 3 show the first stage of the Lord–Wingersky algorithm 2.5 to compute the bivariate posterior of $\eta$ and $\xi _{1}$. In Table 2, the within-cluster summed score likelihoods of the three item clusters are computed. In Table 3, specific dimensions $\xi _{2}$ and $\xi _{3}$ are integrated out, leaving the observed summed score of item cluster 2 and 3 as a function of $\eta$. Table 4 shows the second stage of the algorithm, where item clusters 2 and 3 are treated as polytomous items (both with 3 categories), while the rest score likelihoods for item cluster 1 are calculated. Table 5 shows how the bivariate posteriors associated with each summed vs. rest score pattern are computed. Summaries of the bivariate normal approximations of posteriors associated with the score combinations are shown in Table 6.

**Fig. 1:**
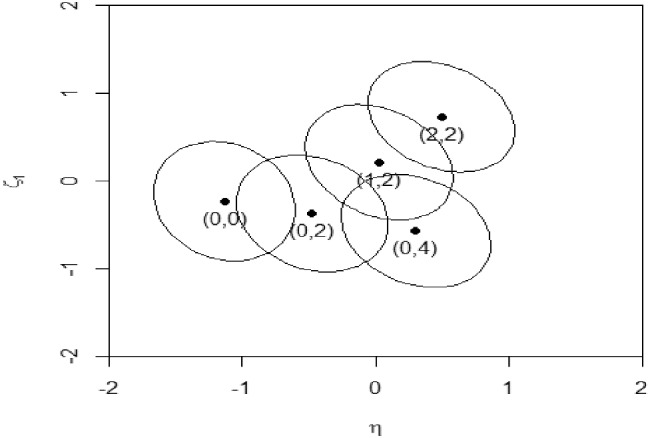
Normal approximations to the posteriors of $\eta$ and $\xi _{1}$ for five score combinations

Figure 1 shows the equal probability contours of the bivariate normal approximated posteriors for five score combinations (with the mean vectors and covariance matrices in Table 6). Each contour includes 25% of the volume under the posterior density. The five score combinations $\left( s_{\mathrm {1}}\mathrm {,}\,s_{(1)} \right)$ are (0, 0$\mathrm {)}$, (0, 2$\mathrm {)}$, (0$\mathrm {, }$4$\mathrm {)}$, (1$\mathrm {, }$2$\mathrm {)}$ and (2$\mathrm {, }$2$\mathrm {)}$, and the corresponding posterior means of $\xi _{\mathrm {1}}$ are: − .232, − .370, − .573, .212, and .732, and those of $\eta$ are: − 1.136, − .477, .302, .025, and .492.

**Table 6:** Summaries of posteriors associated with each score combination

	Prob.	$\mu _{\mathrm {0}}$	$\sigma _{00}$	$\mu _{1}$	$\sigma _{11}$	$\sigma _{01}$
$s_{\mathrm {1}}\mathrm {=0, }s_{(1)}\mathrm {=0}$	.054	$-$ 1.136	.488	$-$ .232	.815	$-$ .091
$s_{\mathrm {1}}\mathrm {=1, }s_{(1)}\mathrm {=0}$	.019	$-$ .640	.528	.413	.756	$-$ .148
$s_{\mathrm {1}}\mathrm {=2, }s_{(1)}\mathrm {=0}$	.005	$-$ .168	.513	.930	.640	$-$ .150
$s_{\mathrm {1}}\mathrm {=0, }s_{(1)}\mathrm {=1}$	.111	$-$ .812	.540	$-$ .296	.796	$-$ .113
$s_{\mathrm {1}}\mathrm {=1, }s_{(1)}\mathrm {=1}$	.053	$-$ .304	.533	.315	.754	$-$ .162
$s_{\mathrm {1}}\mathrm {=2, }s_{(1)}\mathrm {=1}$	.019	.162	.519	.832	.664	$-$ .156
$s_{\mathrm {1}}\mathrm {=0, }s_{(1)}\mathrm {=2}$	.146	$-$ .477	.560	$-$ .370	.775	$-$ .131
$s_{\mathrm {1}}\mathrm {=1, }s_{(1)}\mathrm {=2}$	.096	.025	.536	.212	.753	$-$ .172
$s_{\mathrm {1}}\mathrm {=2, }s_{(1)}\mathrm {=2}$	.046	.492	.527	.732	.688	$-$ .159
$s_{\mathrm {1}}\mathrm {=0, }s_{(1)}\mathrm {=3}$	.110	$-$ .091	.552	$-$ .466	.750	$-$ .144
$s_{\mathrm {1}}\mathrm {=1, }s_{(1)}\mathrm {=3}$	.101	.392	.531	.092	.752	$-$ .177
$s_{\mathrm {1}}\mathrm {=2, }s_{(1)}\mathrm {=3}$	.067	.850	.511	.624	.711	$-$ .151
$s_{\mathrm {1}}\mathrm {=0, }s_{(1)}\mathrm {=4}$	.050	.302	.545	$-$ .573	.725	$-$ .155
$s_{\mathrm {1}}\mathrm {=1, }s_{(1)}\mathrm {=4}$	.064	.771	.519	$-$ .036	.751	$-$ .175
$s_{\mathrm {1}}\mathrm {=2, }s_{(1)}\mathrm {=4}$	.059	1.205	.456	.521	.731	$-$ .131

Examining the means and variances reveals some interesting patterns. First, all the posterior covariances between the primary and specific dimension are negative, even when they are *a priori* uncorrelated. Second, when the rest score remains the same (2), the specific dimension score increases from $-$ .370 to .212 and .732 as the summed score for item cluster 1 increases from 0 to 1 and 2, which is to be expected. Third, because the total summed score for the combination ($0\mathrm {, }4\mathrm {)}$ is 4, and for the combination (1, 2) is 3, we intuit that the second combination should imply a lower primary dimensional score (.302 vs. .025), as expected. Fourth, holding item cluster 1 score constant (0), the primary dimension score is increasing (from $-$ 1.136 to $-$ .477 and to .302$\mathrm {)}$ as the rest score moves from 0 to 1 and 2, again as expected. Fifth, the posterior means of the specific dimension, on the other hand, decreases from $-$ .232, to $-$ .370, and ultimately to $-$ .573, consistent with the negative posterior correlations between the primary and specific dimensions. In this case, the posterior variance shrinks from .815 to .775 to .725, indicating increasing certainty in the specific dimension score. Finally, the posterior variance for the general dimension is .536 for the score combination (1, 2). This is slightly smaller than the reported posterior variance (.55) of the general dimension in Cai (2015) for same total score (3), because the latter variance is conditional on the total summed score alone, a further reduction of the observed data.

### The More General Case

The algorithm generalizes naturally when the number of general dimensions exceeds 1 $(M>1)$. In this case, the single set of rectangular quadrature points $Y_{q}$ that cover the latent variable space of $\varvec{\eta }$ becomes a direct product of *M* sets of quadrature points. The marginal posterior means $\varvec{\mu }_{0}=E\left( \varvec{\eta }\vert s_{n}, s_{(n)} \right)$ is now a $M\times 1$ vector, the primary dimension error covariance matrix ${\varvec{\Sigma }}_{00}= Var\left( \varvec{\eta }\vert s_{n}, s_{(n)} \right)$ is $M\times M$, and the covariance terms in $\varvec{\sigma }_{0n}=Cov\left( \varvec{\eta },\xi _{n}\vert s_{n}, s_{(n)} \right)$ become a $M\times 1$ vector.

It is worth noting if there are more than two item clusters, the estimates of $\varvec{\eta }$ will change depending on which item cluster is treated as the focal cluster (e.g., cluster 1 vs. the rest, or cluster 2 vs. the rest, etc.). This phenomenon is analogous to the difference between response pattern-based scaled scores and summed scores-based scaled scores in unidimensional IRT models that do not assume equal item slope parameters. Items with varying slope parameters are not equally discriminating, and different response patterns with the same summed score will necessarily lead to different scaled score estimates. This is well understood. In hierarchical item factor models, item clusters take the place of items. The item clusters may have different difficulty and discriminability as far as $\varvec{\eta }$ is concerned, and therefore different ways of decomposing the total summed score will lead to different $\varvec{\eta }$ estimates. For a reader whose only concern is scoring for $\varvec{\eta }$, Cai’s (2015) algorithm may be simpler and more easily interpretable, though of course, cluster score combinations do condition on varied patterns of responses and contain more information than the total score.

## Illustrative Applications of Lord–Wingersky Algorithm 2.5

### Growth Interpretation of Observed Score Combinations

ELPA21 is a multi-state assessment program that provides measures of English language proficiency of English Learners (ELs) in K-12 educational systems in the participating states. It measures ELs’ proficiencies in four language domains—reading, listening, writing, and speaking—from kindergarten to high school (i.e., kindergarten, grade 1, grade band 2–3, grade band 4–5, grade band 6–8, and grade band 9–12). Cut scores were established from standard setting studies in each domain and grade band so that students are classified as *emerging*, *progressing*, or *proficient*. Parameters of items in tests of different grade bands were calibrated separately (CRESST, 2017). Thus, the latent ability scales of two adjacent grade bands are different and the scale scores of tests of different grade bands cannot be compared directly.

A convenient and transparent way to report students’ growth is desired, but ELPA21 took the position that vertical scaling, a technique popular in statewide accountability testing, should not employed. The major reason for the choice was that language learning and proficiency development change rapidly over time, especially in the early stage (e.g., PK to 2), potentially shifting the construct being measured considerably. Measures of proficiency such as ELPA21 test scores probably should not be compared directly (or forced on the same scale) for a PK English Learner vs. an English Learner in upper elementary. Hansen and Monroe (2018) provide additional discussions of this topic. Here, we explore the possible use of observed score combinations to describe student growth through the application of Lord–Wingersky algorithm 2.5.

Consider two fixed-form tests of in adjacent grade bands—one in the lower-grade band and one in the upper-grade band. The example here is the listening tests from grade bands 4–5 and 6–8. The lower-grade band test consists of 24 dichotomous items, and upper-grade band test includes 30 dichotomous items. A random sample of 300 students who took both tests was used to estimate the population distribution, while item parameters were held at the pre-calibrated values (see Table 7). Items in each test form are thought to load on either the lower- or upper-grade band listening proficiency latent variables, depending on which form they come from. The two latent variables are correlated, resulting in a classical correlated-traits MIRT model for longitudinal data. The estimated population means of the lower- and upper-grade band latent proficiency variables is .09 (${\text {SE}} = .08$) and $-$ .05 (${\text {SE}} = .06$), respectively. Estimated population variances of the two grade bands are 1.25 (${\text {SE}} = .15$) and .62 (${\text {SE}} = .07$), and their covariance is .80 (SE $=$ .09), yielding a correlation of .91.

**Table 7:** Item parameters of the ELPA 21 test forms in two consecutive years

Grade band	Item ID	Intercept	Slopes			
			$\eta _{1}$	$\eta _{2}$	$\xi _{1}$	$\xi _{2}$
Lower	1	3.26	1.43	0.00	0.00	
Lower	2	2.95	1.53	0.00	0.00	
Lower	3	1.10	0.46	0.00	0.00	
Lower	4	2.85	1.88	0.00	0.00	
Lower	5	1.95	1.51	0.00	0.00	
Lower	6	1.59	1.10	0.00	0.00	
Lower	7	2.82	1.50	0.00	0.00	
Lower	8	4.02	1.64	0.00	0.00	
Lower	9	0.18	0.29	0.00	0.00	
Lower	10	2.08	1.27	0.00	0.00	
Lower	11	2.24	1.28	0.00	0.00	
Lower	12	1.70	0.95	0.00	0.00	
Lower	13	4.34	1.71	0.00	0.00	
Lower	14	2.80	1.52	0.00	0.00	
Lower	15	3.77	2.10	0.00	0.00	
Lower	16	2.96	1.80	0.00	0.00	
Lower	17	3.33	1.79	0.00	0.00	
Lower	18	0.33	0.76	0.00	0.00	
Lower	19	$-$ 0.95	0.57	0.00	0.00	
Lower	20	2.18	1.46	0.00	0.00	
Lower	21	1.79	1.20	0.00	0.00	
Lower	22	1.78	1.57	0.00	0.00	
Lower	23	2.49	1.65	0.00	0.00	
Lower	24	1.38	1.01	0.00	0.00	
Upper	1	1.60	0.00	1.48		0.00
Upper	2	0.98	0.00	1.54		0.00
Upper	3	2.34	0.00	1.73		0.00
Upper	4	1.65	0.00	1.42		0.00
Upper	5	2.20	0.00	1.64		0.00
Upper	6	0.89	0.00	1.10		0.00
Upper	7	2.94	0.00	2.03		0.00
Upper	8	2.70	0.00	1.32		0.00
Upper	9	5.40	0.00	2.64		0.00
Upper	10	3.51	0.00	2.24		0.00
Upper	11	5.40	0.00	2.73		0.00
Upper	12	4.34	0.00	2.16		0.00
Upper	13	4.09	0.00	2.14		0.00
Upper	14	6.03	0.00	2.04		0.00
Upper	15	5.76	0.00	2.95		0.00
Upper	16	4.94	0.00	2.10		0.00
Upper	17	4.71	0.00	2.56		0.00
Upper	18	7.92	0.00	2.95		0.00
Upper	19	2.67	0.00	1.66		0.00
Upper	20	2.45	0.00	1.81		0.00
Upper	21	0.66	0.00	1.49		0.00
Upper	22	2.14	0.00	1.74		0.00
Upper	23	1.51	0.00	1.81		0.00
Upper	24	1.93	0.00	1.39		0.00
Upper	25	2.51	0.00	1.90		0.00
Upper	26	2.72	0.00	1.86		0.00
Upper	27	3.85	0.00	2.31		0.00
Upper	28	0.35	0.00	0.73		0.00
Upper	29	2.28	0.00	1.65		0.00
Upper	30	1.39	0.00	1.26		0.00

**Table 8:** Two-way lookup table that translates observed subscore combinations to composite scores

Listening score	Reading score																
	0	1	2	3	4	$\ldots$	9	10	11	12	13	$\ldots$	19	20	21	22	23
0	$-$ 3.96	$-$ 3.81	$-$ 3.65	$-$ 3.50	$-$ 3.35	$\ldots$	$-$ 2.71	$-$ 2.59	$-$ 2.46	$-$ 2.35	$-$ 2.24	$\ldots$	$-$ 1.75	$-$ 1.69	$-$ 1.64	$-$ 1.60	$-$ 1.56
1	$-$ 3.77	$-$ 3.62	$-$ 3.47	$-$ 3.33	$-$ 3.19	$\ldots$	$-$ 2.59	$-$ 2.48	$-$ 2.36	$-$ 2.26	$-$ 2.15	$\ldots$	$-$ 1.68	$-$ 1.62	$-$ 1.57	$-$ 1.53	$-$ 1.49
2	$-$ 3.58	$-$ 3.44	$-$ 3.30	$-$ 3.17	$-$ 3.04	$\ldots$	$-$ 2.48	$-$ 2.37	$-$ 2.27	$-$ 2.17	$-$ 2.07	$\ldots$	$-$ 1.61	$-$ 1.55	$-$ 1.50	$-$ 1.45	$-$ 1.42
3	$-$ 3.40	$-$ 3.27	$-$ 3.14	$-$ 3.02	$-$ 2.90	$\ldots$	$-$ 2.38	$-$ 2.28	$-$ 2.18	$-$ 2.08	$-$ 1.99	$\ldots$	$-$ 1.54	$-$ 1.48	$-$ 1.43	$-$ 1.39	$-$ 1.35
4	$-$ 3.24	$-$ 3.12	$-$ 3.00	$-$ 2.88	$-$ 2.78	$\ldots$	$-$ 2.29	$-$ 2.19	$-$ 2.10	$-$ 2.01	$-$ 1.92	$\ldots$	$-$ 1.47	$-$ 1.41	$-$ 1.36	$-$ 1.32	$-$ 1.28
5	$-$ 3.10	$-$ 2.98	$-$ 2.87	$-$ 2.76	$-$ 2.66	$\ldots$	$-$ 2.20	$-$ 2.11	$-$ 2.02	$-$ 1.93	$-$ 1.85	$\ldots$	$-$ 1.41	$-$ 1.35	$-$ 1.30	$-$ 1.26	$-$ 1.22
$\ldots$	$\ldots$	$\ldots$	$\ldots$	$\ldots$	$\ldots$	$\ldots$	$\ldots$	$\ldots$	$\ldots$	$\ldots$	$\ldots$	$\ldots$	$\ldots$	$\ldots$	$\ldots$	$\ldots$	$\ldots$
10	$-$ 2.52	$-$ 2.43	$-$ 2.34	$-$ 2.27	$-$ 2.19	$\ldots$	$-$ 1.84	$-$ 1.76	$-$ 1.69	$-$ 1.61	$-$ 1.54	$\ldots$	$-$ 1.10	$-$ 1.05	$-$ 1.00	$-$ .95	$-$ .91
11	$-$ 2.43	$-$ 2.34	$-$ 2.26	$-$ 2.19	$-$ 2.12	$\ldots$	$-$ 1.77	$-$ 1.70	$-$ 1.62	$-$ 1.55	$-$ 1.48	$\ldots$	$-$ 1.05	$-$ .99	$-$ .94	$-$ .90	$-$ .86
12	$-$ 2.34	$-$ 2.26	$-$ 2.18	$-$ 2.11	$-$ 2.04	$\ldots$	$-$ 1.70	$-$ 1.63	$-$ 1.56	$-$ 1.49	$-$ 1.42	$\ldots$	$-$ 1.00	$-$ .94	$-$ .89	$-$ .85	$-$ .81
13	$-$ 2.25	$-$ 2.17	$-$ 2.10	$-$ 2.03	$-$ 1.96	$\ldots$	$-$ 1.64	$-$ 1.57	$-$ 1.50	$-$ 1.44	$-$ 1.37	$\ldots$	$-$ .95	$-$ .89	$-$ .84	$-$ .79	$-$ .75
14	$-$ 2.17	$-$ 2.09	$-$ 2.02	$-$ 1.96	$-$ 1.89	$\ldots$	$-$ 1.59	$-$ 1.52	$-$ 1.46	$-$ 1.39	$-$ 1.33	$\ldots$	$-$ .88	$-$ .82	$-$ .76	$-$ .72	$-$ .67
15	$-$ 2.09	$-$ 2.02	$-$ 1.96	$-$ 1.90	$-$ 1.84	$\ldots$	$-$ 1.54	$-$ 1.47	$-$ 1.41	$-$ 1.34	$-$ 1.27	$\ldots$	$-$ .80	$-$ .74	$-$ .68	$-$ .63	$-$ .59
$\ldots$	$\ldots$	$\ldots$	$\ldots$	$\ldots$	$\ldots$	$\ldots$	$\ldots$	$\ldots$	$\ldots$	$\ldots$	$\ldots$	$\ldots$	$\ldots$	$\ldots$	$\ldots$	$\ldots$	$\ldots$
21	$-$ 1.76	$-$ 1.70	$-$ 1.64	$-$ 1.58	$-$ 1.52	$\ldots$	$-$ 1.23	$-$ 1.17	$-$ 1.10	$-$ 1.03	$-$ .95	$\ldots$	$-$ .34	$-$ .25	$-$ .17	$-$ .10	$-$ .04
22	$-$ 1.70	$-$ 1.64	$-$ 1.58	$-$ 1.53	$-$ 1.47	$\ldots$	$-$ 1.18	$-$ 1.11	$-$ 1.04	$-$ .96	$-$ .87	$\ldots$	$-$ .20	$-$ .10	$-$ .01	.07	.14
23	$-$ 1.65	$-$ 1.59	$-$ 1.53	$-$ 1.47	$-$ 1.41	$\ldots$	$-$ 1.11	$-$ 1.04	$-$ .97	$-$ .89	$-$ .79	$\ldots$	$-$ .04	.07	.17	.26	.34
24	$-$ 1.59	$-$ 1.53	$-$ 1.47	$-$ 1.41	$-$ 1.35	$\ldots$	$-$ 1.04	$-$ .97	$-$ .89	$-$ .81	$-$ .71	$\ldots$	.14	.26	.38	.48	.57
25	$-$ 1.53	$-$ 1.46	$-$ 1.40	$-$ 1.34	$-$ 1.28	$\ldots$	$-$ .97	$-$ .90	$-$ .82	$-$ .73	$-$ .62	$\ldots$	.34	.48	.61	.72	.83
26	$-$ 1.46	$-$ 1.40	$-$ 1.33	$-$ 1.27	$-$ 1.22	$\ldots$	$-$ .90	$-$ .83	$-$ .74	$-$ .64	$-$ .53	$\ldots$	.57	.72	.87	1.00	1.12
27	$-$ 1.40	$-$ 1.33	$-$ 1.27	$-$ 1.21	$-$ 1.16	$\ldots$	$-$ .83	$-$ .75	$-$ .67	$-$ .56	$-$ .43	$\ldots$	.82	.99	1.15	1.30	1.43

**Fig. 2:**
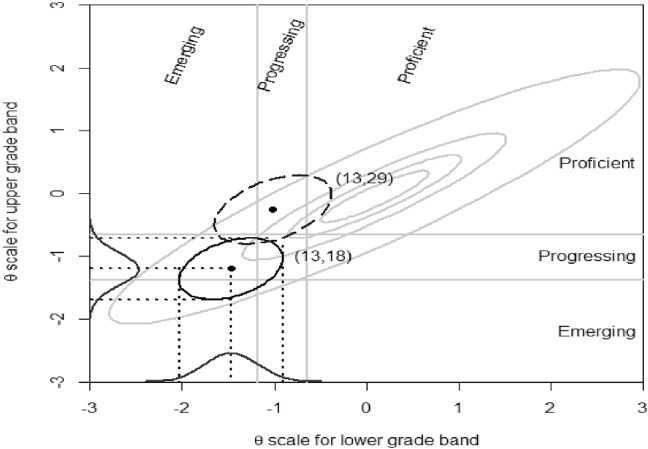
MIRT model to aid ELPA21 growth score interpretation

The x-axis of Fig. 2 is the latent proficiency scale of the lower-grade band, and the y-axis is the latent proficiency scale of the upper-grade band. The estimated (bivariate normal) population distribution is overlaid in light gray. Four cut scores—two for the lower-grade band ($-$ 1.1875 and $-$ 0.65) and two for the upper-grade band ($-$ 1.375 and $-$ 0.65)—divide the space into nine regions. Bivariate normal approximations of posteriors associated with two score combinations are plotted. The two-dimensional MIRT model is treated as a two-tier model with empty specific latent dimensions (no item loading) so that the score combination posteriors can be computed via the Lord–Wingersky algorithm 2.5 implemented in flexMIRT$^{{\circledR }}$ (Cai, 2017) without additional programming. This is analogous to Cai’s (2015) Example 4.2 that replicates more specialized score combination computations, wherein the bifactor model was strictly not needed. The classification of *emerging*, *progressing*, or *proficient *are made out of the volume of the marginal posterior distribution that falls between the cut scores. For example, the probabilities of *emerging*, *progressing*, or *proficient *of students with observed score combination (13, 18) are .84, .16, and 0 in the lower-grade band and .24, .75, and .01 in the upper-grade band. We may then communicate clearly to the users of the score reports that this particular combination of 13 (out of 24) on the lower-grade band test and 18 (out of 30) on the upper-grade band test indicates an improvement from *emerging* to *progressing*. In a similar fashion, the score combination (13, 29) represents an improvement from *progressing* to *proficient*.

The probabilities of each of the combinations are also natural by-products of our recursive algorithm. Among students who received a score of 13 on the lower-grade band test (expected to be roughly 1.89% of the student population, based on the model), a score of 24 on the upper-grade band test places the student at the 74% percentile, which is akin to a student growth percentile (SGP; Betebenner, 2009) but entirely based on observed scores. In addition, although not pursued here, the Lord–Wingersky algorithm 2.5, coupled with the calibrated projection method (Thissen et al., 2011), can be applied to predict scores of the upper grade-band test based on the lower-grade band test scores. In sum, Lord–Wingersky algorithm 2.5 serves as a useful tool to facilitate reporting of student growth in the multi-state EL assessment program.

### Facilitating Subscore Reporting

Educational and psychological assessments usually consist of several item clusters, yielding the so-called subscores. Within the IRT framework, several subscoring approaches, including the bifactor model approach and the correlated-traits MIRT model approach, are available. Subscore reporting is another recurrent topic in recent psychometrics literature (e.g., Sinharay et al., 2007; Haberman, 2008; Haberman et al. 2009; Feinberg & Wainer, 2014) because of the increasing demand for more detailed information about individuals. Two issues must be considered when deciding whether to report subscores obtained through a bifactor model (i.e., the $\xi _{n}$ estimates) in addition to the overall score (i.e., the $\eta$ estimate). The first question—if these subscores are reliable enough—is the easier one to address within the IRT framework. Here we focus on the second question—whether the information the subscores provide is distinct enough from the overall score.

We believe that if a subscore is considered to be surprising given an individual’s overall score (i.e., if the $\xi _{n}$ estimate cannot be well predicted by the $\eta$ estimate), it should be reported for it is adding information. This is similar in spirit to Feinberg and von Davier’s (2020) idea of identifying unexpectedly high or low subscores by comparing observed subscores against a discrete distribution of subscores conditional on the overall proficiency variable in a unidimensional IRT model, but the computations and approach are different.

Our context is a psychiatric assessment tool—the Psychiatric Diagnostic Screening Questionnaire (PDSQ; Zimmerman & Mattia, 2001). PDSQ is a widely used self-report instrument. In particular, it is used in the well-known Sequenced Treatment Alternatives to Relieve Depression (STAR*D) trial, a federally funded large-scale study comparing depression treatments. The instrument consists of 139 dichotomous items that cover 15 most prevalent DSM-IV (American Psychiatric Association, 1994) Axis I disorders. Using STAR*D data, which we also use here, Gibbons et al. (2009) showed that a bifactor model, which includes a general psychiatric distress dimension and 15 domain-specific latent dimensions, provides a plausible theoretical and statistical structure for the instrument.

In our preliminary analysis, three symptom domains—alcohol abuse dependence (ALC), drug abuse dependence (DRUG), and Psychosis (PSYCH)—are excluded. The exclusion of the first two is based on the empirical observation that the substance abuse domains were rather distinct from the other domains, as judged from the item slopes. The Psychosis domain is excluded because the STAR*D participants are screened positive for non-psychotic major depressive disorder. Two more items in the major depressive disorder (MDD) domain were further excluded due to their ill fit. Therefore, the MDD domain is measured by 19 dichotomous items, while the rest score on the other 11 domains can range from 0 to 100. Item parameters are calibrated based on a sample of 3999 participants and are assumed to be fixed in the subsequent analysis. The illustrative task is to identify combinations of observed scores on the MDD domain (i.e., $$s_{1})$$ versus the rest (i.e., $$s_{(1)})$$ that signal the reporting of the MDD subscore would add information to the overall score.

**Fig. 3:**
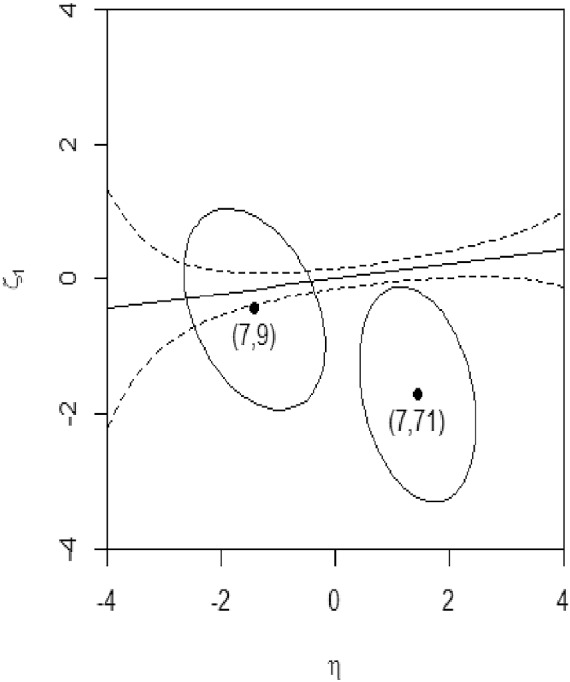
$95{\%}$ prediction interval of $\eta$-$\xi _{1}$ regression

We note that the summaries of the posterior distribution (i.e., $\varvec{\mu }$ and ${\varvec{\Sigma }})$ along with the probability associated with each observed score combination, obtainable from the Lord–Wingersky algorithm 2.5, could be utilized to capture the statistical relationship between $\xi _{n}$ and $\eta$ and thereafter facilitate subscore reporting. In the simplest instantiation, we regress the estimate of $\xi _{1}$ of each score combination $(s_{1},s_{(1)})$ on the $\eta$ estimate, weighted by the corresponding marginal probability, $p\left( s_{1},s_{\left( 1 \right) } \right)$. A 95% prediction interval from this weighted least squares regression can be calculated (Fig. 3) with the regression parameter estimates and serves as the basis to evaluate the bivariate normal approximated posterior of each $(s_{1},s_{(1)})$. For each score combination, the proportion of the posterior density volume that falls within the prediction interval is computed, akin to a *p*-value. The smaller the proportion, the more necessity there is to report the $\xi _{1}$ estimate associated with the score combination. For example, as in Fig. 3, the MDD subscore associated with (7, 71$\mathrm {)}$ should be reported, while for another combination, (7, 9$\mathrm {)}$, it may not be necessary. Table 9 shows proportions of posterior volumes that fall in the prediction interval, with darker cells indicating lower proportions.

**Table 9:**
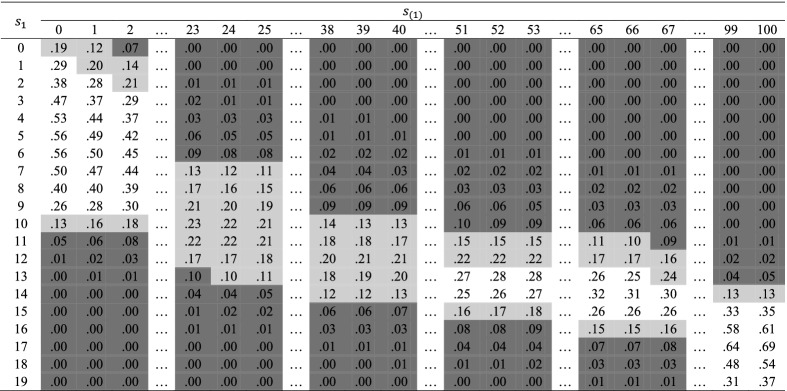
Proportions of posterior volume that falls in prediction interval

### Detecting Aberrant Score Combination Pattern

As mentioned in Sect. 4.1, the probability of each observed score combination is a by-product of the Lord–Wingersky algorithm 2.5. When arranged in a contingency table, the probability of observed subscore combination $(s_{n},s_{(n)})$ can be used to detect aberrant score combinations through the construction of posterior high-density region (HDR; Novick & Jackson, 1974). A low probability indicates that the co-occurrence of corresponding summed scores is rare. Depending on context, this approach can be useful for diagnosis of lack of person fit or for forensic data analysis in test security.

We illustrate this application of Lord–Wingersky algorithm 2.5 with the Quality of Life (QoL) Scale for the Chronically Mentally Ill (Lehman, 1988). Many previous studies indicate a bifactor model fits the 35-item QoL scale extremely well (e.g., Gibbons et al., 2007; Cai & Hansen, 2013). Beyond an overall quality of life item, there are 7 subscales (*Family*, *Finance*, *Health*, *Leisure*, *Living*, *Safety*, and *Social*), each of which includes 4 to 6 items. The dataset used here includes responses from 586 patients. To aid presentation, the original 7-category rating scale items are recoded to have two categories (i.e., 0, 1, and 2 in the original scale are recoded as 0; 3, 4, 5, and 6 as 2). Here, we construct the high-density region (HDR) of combinations of the score on *Health *($$s_{1})$$ and the rest score ($$s_{(1)})$$ using the Lord–Wingersky algorithm 2.5. $$s_{1}$$ ranges from 0 to 6, and $$s_{(1)}$$ ranges from 0 to 29.

To construct a HDR of level $\alpha$, we first stack the $p(s_{1},s_{(1)})$ of each score combination into a single column, sort all the probabilities from the largest to the smallest, and then compute the cumulative distribution of these probabilities. Observed score combinations that contribute to the first $100\alpha \%$ of the cumulative distribution are identified as the $100\alpha \%$ HDR.

**Fig. 4:**
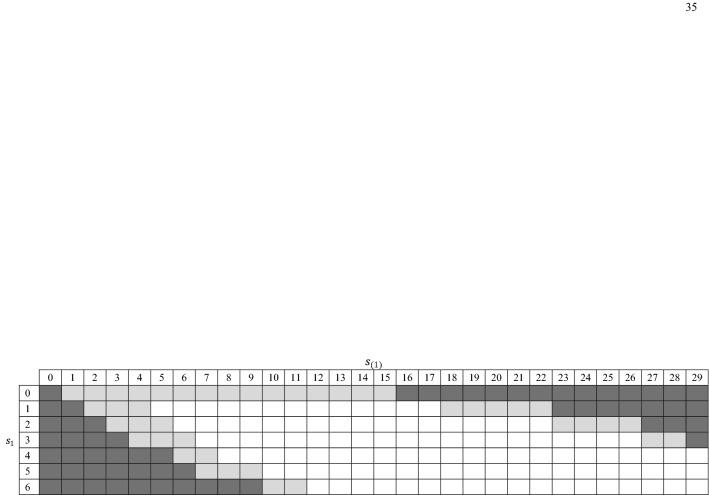
High-density region (*Health* versus the rest)

Figure 4 shows the HDR for the illustrative task. The unshaded cells represent the 95% HDR. The light gray cells together with the unshaded cells represent the 99% HDR. The dark gray cells represent observed subscore patterns that rarely occur. For example, the score combinations (0, 29) and (6, 0) rarely occur. Individuals with such score combinations deserve further attention.

## Discussion

The original Lord–Wingersky (1984) algorithm was developed for binary items under unidimensional IRT models. Then the algorithm was expanded to polytomous unidimensional IRT models (Hanson, 1994; Thissen et al., 1995; von Davier & Rost, 1995). The Lord–Wingersky algorithm version 2.0 (Cai, 2015) was proposed to computed likelihoods associated with overall summed scores in the context of hierarchical item factor models. In the present article, we proposed the Lord–Wingersky algorithm 2.5 as an extension of the Cai’s (2015) Lord–Wingersky algorithm 2.0. The algorithm yields the characterization of the bivariate posterior associated with observed score combinations from the mutually exclusive clusters of items in the model. The algorithm uses more observed information than the Lord–Wingersky Algorithm 2.0 (observed score combinations instead of one overall summed score). Thus it can provide additional information that is useful in practice (summed score likelihoods for all latent dimension instead of the likelihood for the primary latent dimension only). The Lord–Wingersky algorithm 2.5 also remains computationally efficient due to the continued use of dimension reduction. With the Lord–Wingersky algorithm 2.5, likelihoods of observed score combinations under several IRT models, including the two-tier model, the bifactor model and the standard MIRT model, can be computed directly under one algorithm.

The bivariate normal approximation (summarized by $\varvec{\mu }$ and ${\varvec{\Sigma }})$ to the posterior associated with each observed score combination, as one of the outputs of the Lord–Wingersky algorithm 2.5, is a reasonable alternative to the actual (intractable) posterior distribution and can serve multiple purposes in educational and psychological measurement. The marginal probability of each observed score combination, which comes as a by-product of the proposed algorithm, is also useful in practice. We use three empirical applications to illustrate the range of possible use of this new algorithm—(a) translating observed score combinations to aid growth interpretations in educational measurement, (b) facilitating subscore reporting in psychiatric assessment, and (c) detecting aberrant observed subscore combinations in health-related outcome research.

While applying the proposed algorithm, we assume the IRT model is correct. It is also assumed that item parameters are known and fixed, since in practice the parameter calibration stage and scoring stage are often conducted sequentially. To take into account the uncertainty around item parameters (i.e., standard errors in the calibration stage), we suggest using multiple imputation (MI; Rubin, 1987)-based approach (e.g., Yang et al., 2012).

Hierarchical item factor models, especially the bifactor model, saw increasing use in psychological and educational assessment. Recent development in computational algorithms for estimating multidimensional IRT models (Cai, 2010a; Edwards, 2010) and software, e.g., flexMIRT$^{{\circledR }}$ (Cai, 2017), has brought the usage of MIRT models within reach for routine data analysis. We posit that providing scores that are based on observed statistics (e.g., summed scores, observed subscale scores) will continue to be desired and useful in practice, and the current study is a further contribution to the IRT scoring literature.
